# Direct Current Electrical Fields Improve Experimental Wound Healing by Activation of Cytokine Secretion and Erk1/2 Pathway Stimulation

**DOI:** 10.3390/life11111195

**Published:** 2021-11-05

**Authors:** Chao Lu, Jonas Kolbenschlag, Andreas K. Nüssler, Sabrina Ehnert, Colin D. McCaig, Urška Čebron, Adrien Daigeler, Cosima Prahm

**Affiliations:** 1 Department of Hand-, Plastic, Reconstructive and Burn Surgery, BG Trauma Clinic Tuebingen, University of Tuebingen, Schnarrenbergstr. 95, D-72076 Tuebingen, Germany; chao.lu@student.uni-tuebingen.de (C.L.); jkolbenschlag@bgu-tuebingen.de (J.K.); UCebron@bgu-tuebingen.de (U.Č.); adaigeler@bgu-tuebingen.de (A.D.); 2 Siegfried Weller Research Institute, Department of Trauma and Reconstructive Surgery, BG Trauma Clinic Tuebingen, University of Tuebingen, Schnarrenbergstr. 95, D-72076 Tuebingen, Germany; andreas.nuessler@med.uni-tuebingen.de (A.K.N.); sabrina.ehnert@med.uni-tuebingen.de (S.E.); 3 Institute of Medical Sciences, School of Medicine, Medical Sciences and Nutrition, University of Aberdeen, Aberdeen AB25 2ZD, UK; c.mccaig@abdn.ac.uk

**Keywords:** direct current electrical fields, HaCaT cells, wound healing, electrical stimulation

## Abstract

There is growing evidence that cell behaviors can be influenced by the direct current electric fields (EFs). Some behaviors may influence wound healing directly. This study aimed to investigate the effects of EF (200 mV/mm) on immortalized nontumorigenic human epidermal (HaCaT) cells. We established a setup that can transmit an EF and maintain a stable cell culture environment. An EF was applied to HaCaT cells, and scratch-assays were performed as a model of wound healing to observe cell migration. Proliferation was evaluated by mitochondrial activity, total protein, and DNA content. Secretion of healing-associated cytokines was evaluated via cytokine arrays, and Western blot was applied to investigate signaling pathway alterations. Compared with the control group, the migration of cells exposed to EFs significantly increased (*p* < 0.01). After 7 days, the changes in proliferation also increased significantly (*p* < 0.05). The cytokine arrays revealed that granulocyte-macrophage colony-stimulating factor (GM-CSF) was the most abundant factor secreted by HaCaT following EF exposure. The signals for phospho-Erk1/2 showed a significant (*p* < 0.0001) increase following EF exposure. The results demonstrate that exposure of HaCaT cells to EFs has positive effects on migration, proliferation, and cytokine secretion—three important steps in wound healing—and these effects may be partially mediated by activation of the Erk1/2 signaling pathway.

## 1. Introduction

Wound healing is a multistep biological process that is highly orchestrated and regulated, and it is focused on the recovery of the injured tissue. Wound healing begins at the moment of injury. The damage that disrupts the epithelial layer instantly generates endogenous electric fields (EFs), which were detected in human skin wounds 150 years ago [1] At the moment of tissue injury, a current is generated that triggers elements of the wound-healing process. The basis for this current is the transepithelial potential difference (TEP), which results from the directed transfer of ions by epithelial cells [2]. When a wound disrupts the epithelial barrier, it short-circuits the TEP. As a result, the potential at the wound collapses, becoming negative relative to the healthy epidermis surrounding the wound [3]. The endogenous wound electric fields, which range from 40 to 200 mV/mm [4], are vectors that occur at the wound site immediately after injury and are considered triggers for tissue repair [1].

The use of electrical stimulation for certain diseases has shown excellent clinical results. In a meta-analysis examining the effectiveness of electrical therapy in chronic wound healing, the application of electrical stimulation resulted in an improved healing rate of 22% per week compared with 9% in controls [5]. Furthermore, EFs have been shown to modulate local tissue inflammation and initiate muscle and tendon regeneration [6]. Positive effects of EFs have also been reported in other medical fields, such as pain management [7], the healing of bone fractures [8], and even the treatment of tumors [9].

The mechanisms underlying these clinical effects have been increasingly studied but are not yet fully understood. In the field of bone tissue engineering, for example, the effect of different field strengths ranging from 0.000048 mV/mm to 6000 mV/mm have been applied to investigate functional changes in osteocytes, such as alkaline phosphatase activity and mineral formation [10]. As a similar but more sophisticated biophysical input, a study on EMFs (electromagnetic fields) also demonstrated their strong anti-inflammatory effects on immune cells and uncovered the mechanism behind them [11]. Although considerable knowledge exists about the beneficial effects of EFs in the medical field, little is known about the role of EFs in wound healing in the skin. One of the reasons is that the existing EF stimulation systems focus more on cell morphology or short-term functional changes [12,13,14,15] than on long-term studies. Specifically, devices focus on single-well applications, which makes reproducibility and large-scale applications difficult [16]. Furthermore, the complexity of existing experimental devices reduces the stability of long-term in vitro research; thus, experiments are time-consuming and expensive.

The aim of this study was to design a device that fulfilled the following criteria: 1. applying EF stimulation in vitro and ensuring the stability of the cell culture environment, and 2. addressing the limitations of replication. Thus, this study aimed to create a time- and effort-saving approach for subsequent research. Using this experimental setup, we investigated the effects of an EF (200 mV/mm, similar to that found in mammalian skin wounds [17]) on the migration, proliferation, growth factor secretion, and signaling pathway changes in skin cells, as these are vital factors in wound healing.

As a skin-derived human keratinocyte cell line, HaCaT cells were eligible and widely used candidates for wound healing investigation [18]. Their ability to proliferate and migrate well mimics the key steps in the healing process; moreover, they can sensitively respond to electrical stimuli [14]. Therefore, HaCaT cells were chosen for the in vitro wound-healing model in this study.

## 2. Materials and Methods

### 2.1. The EF Stimulation Setup and EF Application

**Figure 1:**
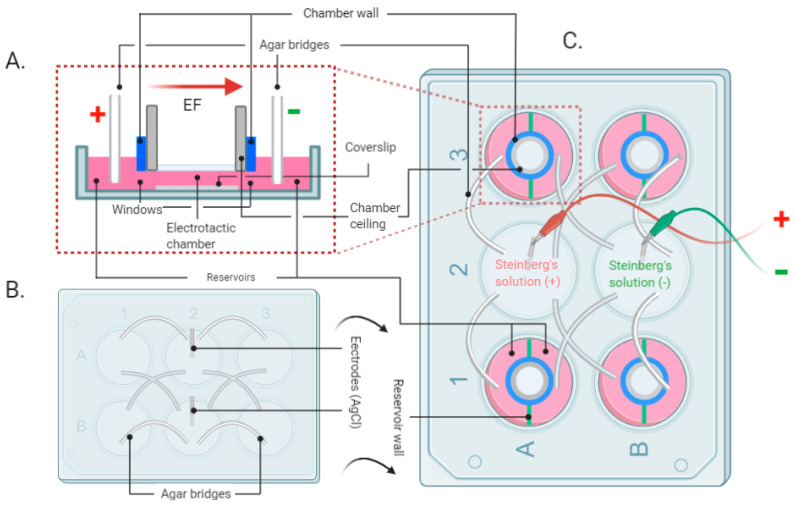
Schematic of the EF stimulation setup developed for in vitro experiments. (**A**) Side view of the EF stimulation zone. (**B**) Scheme of the custom-built lid of the experimental setup. (**C**) Top view of the assembled experimental setup. (Created with BioRender.com 13 October 2021).

The setup for EF stimulation (Figure 1) was adapted to 6-well cell culture plates (Corning Costar, Merck, Darmstadt, Germany) based on a previously described principle [19]. The experimental setup (Figure 1C) consisted of two main elements: a standard 6-well cell culture plate, which holds four identical EF stimulation zones (Figure 1A), and a modified lid of the 6-well plate (Figure 1B). In particular, the newly designed experimental setup can transmit a specific electric field generated by an electrical stimulation device (Vanquish Innovation, New York, NY, USA) and uniformly distributed by saline solutions and agar bridges to four EF stimulation zones in parallel.

The middle row of the 6-well plate (wells A2 and B2) served as the anode (A2) and cathode (B2), whereas the remaining outer wells were used as the EF stimulation zones (A1/3, B1/3). To set up the experiment, wells A2 and B2 of the 6-well plate were filled with a saline solution (Steinberg’s solution), and the four EF stimulation zones were filled with a cell culture medium (Dulbecco’s Modified Eagle Medium, DMEM, Life Technologies, Darmstadt, Germany). Afterward, the lid was mounted to close off the chamber (Figure 1C). Two electrodes were connected to the EF generator, providing a continuous potential difference of 7 V (Figure 1C). Eighteen holes were drilled in the standard 6-well plate lid to transmit the electric potential difference via agar bridges connecting the anode and cathode with each EF stimulation zone. The agar bridges were produced using standard clinical infusion tubes filled with sterile 2% agarose (J.T. Baker, Mansfield, MA, USA) diluted in Steinberg’s solution, transmitting the EF signals as well as isolating harmful substances [20]. The remaining two holes in the middle held the electrodes in place.

The EF stimulation zone was divided into two reservoirs with two plastic strips (green parts in Figure 1C) (Figure 1A). An identical electric potential difference was distributed through the agar bridges from the anode and cathode to the respective reservoirs in each EF stimulation zone. To generate an electric field of 200 mV/mm within each stimulation zone, agar bridges were spaced 35 mm apart (Figure 1A). HaCaT cells were seeded on a round (collagen-coated) coverslip and placed in the electrotactic chamber in the middle of the well. The chamber wall was made of a plastic ring (blue part in Figure 1A,C, made from the cap of a 15 mL Falcon tube), where two windows (Figure 1A) with a size of 2.0 mm high × 5.0 mm wide were cut out on opposite sides to ensure the permeability of the EF and medium.

It is crucial to keep the height of the chamber shallow to maintain a stable voltage difference and eliminate the possibility of any Joule heating effect [19].

Therefore, a chamber ceiling was screwed onto the chamber wall after the cells were housed in the electrotactic chamber (grey part in Figure 1A,C). The chamber ceiling was made of a plastic ring with a smaller diameter and a round glass fixed at the bottom (the material was salvaged from the body of a 15 mL Falcon tube). It maintained the depth of the chamber culture medium (or the medium height above the cells) at 0.5 mm.

Via this structure, each of two agar bridges in an EF stimulation zone would be connected to a reservoir and an anode (well no. A2) or cathode (well no. B2), and the identical electrical field of 200 mV/mm could be transmitted in parallel to the four electrotactic chambers.

### 2.2. Scratch Wound Closure Assay

Traditional methods of the scratch assay (using a pipette tip) may cause cell fragments to be generated and pile up on the edge of the scratch, which may interfere with the accuracy of the experimental results. In this study, a reformed scratch assay with HaCaT cultures was used to estimate the ability of the EF to promote wound closure in vitro. In brief, sterile collagen-coated round coverslips (Thermo Scientific, Braunschweig, Germany) were prepared and placed in a 24-well plate (Corning Costar, Merck, Darmstadt, Germany) prior to experiments. Silicon strips made of self-adhesive silicone tape (Tesa Silikonband, Offenburg, Germany) were used to guarantee cell-free areas. The strips (length = 10.0 mm, width = 0.3 mm) were sterilized in an autoclave and adhered to the coverslips to prevent the entrance of cells. HaCaT cells were seeded in the 24-well plate with the coverslips pre-placed at 5 × 10^4^ cells/cm^2^ in DMEM. After 3 h, coverslips with attached cells were carefully transferred into the electrotactic chambers, and the silicon strips were carefully removed. Cell-free areas were generated without any cell debris. Four milliliters of medium were added to the reservoirs, and the ceilings were then screwed on. The lid with agar bridges and electrodes was capped on the setup (Figure 1a), and the EF (200 mV/mm) stimulation was initiated. At the same time, a blank control group underwent the same procedures without EF stimulation.

Cells were cultured in an incubator (5% CO_2_ at 37 °C, Heraeus 6000 Thermo Fischer Scientific, Langenselbold, Germany), and cell migration was monitored using a Zeiss Observer Z1 microscope (Carl Zeiss, AG, Oberkochen, Germany) with 20× magnification every 12 h over a 36-h incubation period. Three images per scratch were taken to achieve an objective evaluation. Cell-free areas were measured by ImageJ (NIH, Bethesda, MD, USA) and calculated according to the following formula [21]:

(1) $$\mathrm{Wound}\;\mathrm{closure}\;\left(\%\right)=\frac{\mathrm{Cell}-\mathrm{Free}\;\mathrm{Area}\left(0\;h\right)-\;\mathrm{Cell}-\mathrm{Free}\;\mathrm{Area}\left(n\;h\right)}{\mathrm{Cell}-\mathrm{Free}\;\mathrm{Area}\left(0\;h\right)}\times 100\%\;,\;n=0,12,24,36\;h$$

### 2.3. Resazurin Conversion Assay

The resazurin conversion assay was employed to assess mitochondrial activity on days 1, 4, and 7 post-exposure. Briefly, 0.0025% (*w/v*) resazurin solution (Sigma-Aldrich, Darmstadt, Germany) (in DPBS) was added to the HaCaT culture for 60 min at 37 °C, and the fluorescence intensity of converted resorufin was measured (ex/em = 540/590 nm) with a plate reader (Omega, BMG Labtech, Ortenberg, Germany) and corrected to background controls as previously described [22].

### 2.4. Sulforhodamine B (SRB) Staining for Total Protein Content

SRB staining was employed to assess the total protein content as previously reported [23] on days 1, 4, and 7 post-exposure. After fixation with ethanol at −20 °C overnight, 0.4% *w/v* SRB (Sigma-Aldrich, Darmstadt, Germany) (in 1% *v/v* acetic acid) was added to cells for 30 min of staining at room temperature (RT). After washing the cells 4–5 times with 1% acetic acid to remove the unbound dye, 10 mM unbuffered TRIS solution (pH = 10.5, Sigma-Aldrich, Darmstadt, Germany) was used to resolve the bound SRB. Immediately afterward, resolved SRB was quantified photometrically at a wavelength of 565–690 nm.

### 2.5. Quantification of Total DNA

To isolate total DNA, HaCaT cells cultured in electrotactic chambers at different time points (days 1, 4, and 7 after EF exposure) were washed once with PBS and incubated in 200 µL of 50 mM NaOH at 98 °C for 5 min with gentle agitation (250 rpm). Following the incubation, the samples were frozen at −20 °C overnight (at least 12 h). After thawing at room temperature for 30 min, 200 µL of a 100 mM Tris buffer (pH = 8.0) was added to each sample to neutralize the pH. These samples were centrifuged at 14,000× *g* at 4 °C for 10 min to remove the insoluble fragments before supernatants were transferred into fresh reaction tubes. The DNA content was determined photometrically using a FLUOstar Omega plate reader (BMG Labtech, Ortenberg, Germany) with the LVIS Plate (BMG Labtech) [24].

### 2.6. Hoechst and Calcein-AM Staining

Nuclei were identified by Hoechst 33,342 staining (blue fluorescence, Sigma-Aldrich, Darmstadt, Germany). Intracellular esterase activity staining using Calcein-AM (green fluorescence, Sigma-Aldrich, Darmstadt, Germany) was applied to visualize living cells. Briefly, HaCaT cells of the control and experimental groups were washed three times with DPBS at specific experimental time points and incubated with Hoechst 33,342 (1 mg/mL) and Calcein-AM (2 µM) on a plate shaker protected from light for 30 min (RT). Fluorescent images were obtained with an immunofluorescence microscope (Epifluorescence: EVOS FL, Life Technologies, Darmstadt, Germany) [25].

### 2.7. Measurement of Cytokine Expression using the Antibody Array Method

We investigated human growth factor expression at the protein level after 7 days of EF exposure via the RayBio Human Growth Factor Array^®^ (RayBiotech, Norcross, GA, USA), which can detect 41 growth factor proteins. The manufacturer’s protocol was strictly followed. Briefly, supernatants of the EF groups after 7 days of exposure and control groups (n = 5) were collected. Cytokine array membranes were washed with the 1st and 2nd washing buffers and incubated in blocking buffer (room temperature, 2 h). Afterward, membranes were incubated with the supernatant of each group (4 ℃, overnight). Following the incubation, the membrane was rewashed and incubated successively with a primary biotin-labeled antibody (room temperature, 1.5 h) and HRP-streptavidin (room temperature, 1.5 h). To visualize the blot, membranes were subsequently washed with ECL substrate solution (1.25 mM luminol, 0.2 mM p-coumaric acid, 0.03% H_2_O_2_ in 100 mM TRIS, pH 8.5), and a CCD camera was used to detect the chemiluminescent signals. The results were quantified using ImageJ and normalized to the positive control (POS) and negative control (NEG) dots on the membranes [26].

### 2.8. Western Blot

The ice-cold radioimmunoprecipitation assay buffer (RIPA buffer) was used to extract protein from cells grown in the new electrotactic chambers. Protein content was determined using the micro-Lowry assay [27]. For all protein detections, 25 μg of total protein was loaded onto 12.5% sodium dodecyl sulfate-polyacrylamide gel electrophoresis (SDS-PAGE) gel, and protein bands were subsequently transferred onto nitrocellulose membranes (Roth, Karlsruhe, Germany). BSA solution (bovine serum albumin) 5% was used to block the nonspecific binding sites for at least 60 min at room temperature. Following blotting, the blots were incubated with primary antibodies (Cell Signaling, Beverly, USA) diluted 1:1000 in TBS-T (Tris-buffered saline, 0.1% Tween 20) at 4 °C overnight. The membrane was then incubated with the corresponding HRP-conjugated secondary antibody (1:10,000 dilutions) for 2 h at room temperature. After three more washes in TBS-T, membranes were incubated with ECL (enhanced chemiluminescence) substrate solution for 1 min. Chemiluminescent signals of target proteins were captured with a ChemoCam Camera (INTAS Science Imaging, Göttingen, Germany). The densitometric analysis of the signal intensities was evaluated using ImageJ software 1.8.0_172 (NIH, Bethesda, MD, USA) and normalized to the expression levels of GAPDH [28].

### 2.9. Statistics

Data were analyzed by the GraphPad Prism software 9.0.0 (El Camino Real, CA, USA); Results are displayed as means ± SEM, N indicates the number of independent experiments, and n indicates the technical replicates of each experiment. *p* < 0.05 was considered statistically significant.

## 3. Results

### 3.1. Finalizing the EF Experimental System

The newly designed experimental setup simplified the structure and improved the stability of the in-vitro experimental system based on the pioneers’ work [19]. Additionally, it can transmit a specific electric field to four EF stimulation zones in parallel, which means the repeatability of the system improved substantially.

**Figure 2:**
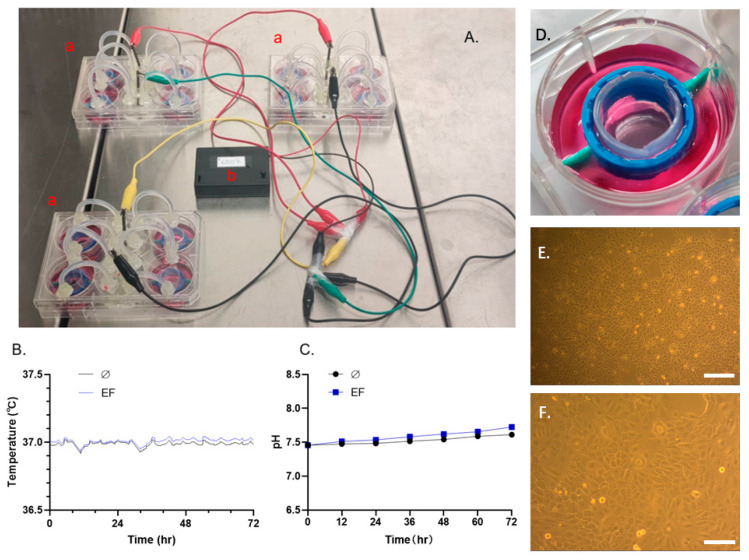
A stable temperature and pH value can be maintained for 72 h in the presence of EF. (**A**) Photograph of the setup during the test: a. well plates with electrodes and agar bridges, b. EF generator. (**B**) The environmental temperature of the EF group did not exceed 0.1 ℃ compared with the control. (**C**) The pH value of the EF group did not exceed 0.2 compared with the control group. (**D**) Image of the EF stimulation zone. (**E**) Micrograph of HaCaT cells cultured in the electrotactic chamber. Magnification 40×, scale bar 200 μm; (**F**) magnification 100×, scale bar 80 μm.

The parameters (pH value and temperature) related to the stability of an in-vitro experimental environment were evaluated. The results showed that the pH value (Figure 2C) and temperature (Figure 2B) could be maintained in the experimental setup for up to 72 h in EF with 200 mV/mm strength, which means a stable culture environment could be maintained between two medium changes.

### 3.2. Effect EF on Scratch Wound Closure in HaCaT cells

**Figure 3:**
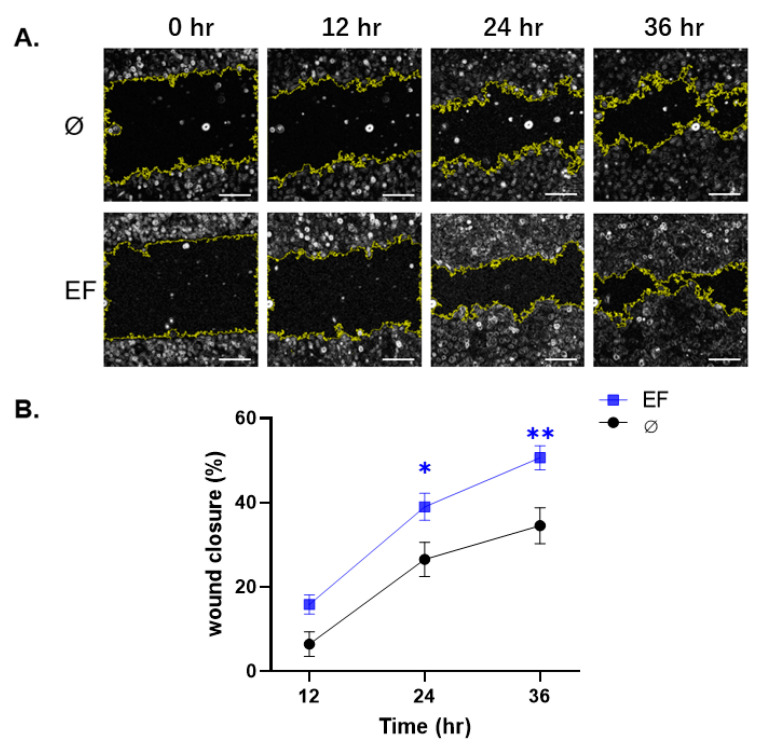
The wound closure speed was increased in HaCaT cells exposed to the EF. The wound closure speed was evaluated after 12, 24, and 36 h of exposure to EF. (**A**) The scratch area was measured by ImageJ automatically. The yellow lines indicate the edge of the scratch. (**B**) The graphs show the difference in wound closure speed, measured as the change in cell-free area over time, assuming the width of the scratch at 0 h was equal to 100%. Scale bar = 100 μm. N = 3, n = 2. Data were compared using non-parametric two-way ANOVA followed by Tukey’s multiple comparison test: * *p* < 0.05, ** *p* < 0.01 compared with the control (no EF).

The influence of EF on HaCaT cell migration in vitro was assessed using a scratch wound assay. The cells were treated in the presence or absence of an EF to quantify the impact of the stimulation on cell wound closure and imaged at defined time points (Figure 3). EF-treated cells showed a significant acceleration of closure speed, which exhibited a ~46.51% improvement relative to the control group 24 and 36 h after exposure (Figure 3B).

### 3.3. EF Application Improves the Proliferation of HaCaT Cells

**Figure 4:**
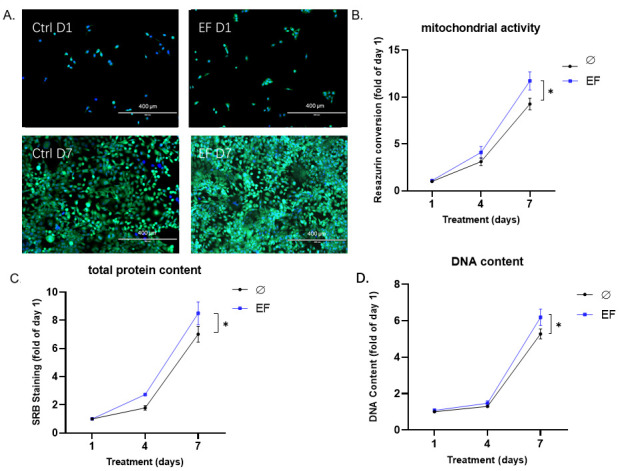
EF application improved the viability and proliferation of HaCaT cells. HaCaT cells were exposed to an EF of 200 mV/mm. On days 1, 4, and 7 of culture (**A**), Hoechst and Calcein AM staining were performed on day 1 and day 7 to visualize massively increasing numbers of healthy cells. (**B**) Mitochondrial activity was determined by resazurin conversion, (**C**) total protein content was determined by SRB staining, and (**D**) total DNA content was determined by the DNA content assay. N ≥ 3, n = 3. Data were compared using non-parametric two-way ANOVA followed by Tukey’s multiple comparison test: * *p* < 0.05 compared with the control (no EF).

HaCaT cells were exposed to an EF with a strength of 200 mV/mm over 7 days. Mitochondrial activity, total protein content, and total DNA content were determined, and Hoechst and Calcein AM staining were performed on days 1, 4, and 7 of the stimulation (Figure 4). The above-mentioned indicators related to cell viability and proliferation continued to increase throughout the 7-day experimental period with EF application. Compared with the control groups, the total protein content (up to 21.4%, Figure 4C) and total DNA content (up to 17.2%, Figure 4D) on day 7 in the EF groups increased significantly (* *p* < 0.05). Similar to the result of total protein and DNA content, on day 7, the mitochondrial activity of HaCaT cells also showed a significant (* *p* < 0.05) increase (up to 26.7%, Figure 4B) compared with the control group. Hoechst and Calcein AM staining confirmed the same (Figure 4A).

### 3.4. Differential Expression of Growth Factors in HaCaT cells after EF exposure

**Figure 5:**
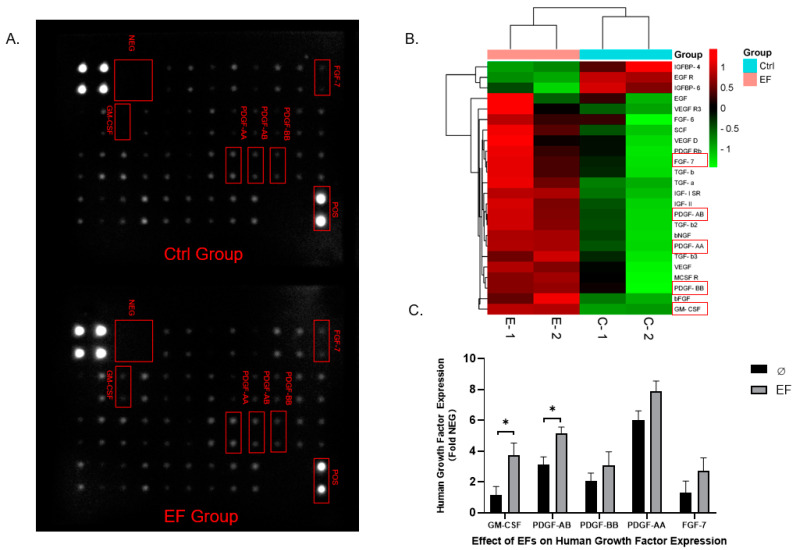
Secretory human growth factor levels in the control group and EF group on day 7. (**A**) The supernatant of HaCaT cells w/wo EF exposure was collected and examined using a growth factor array. POS: positive control; NEG: blank control. (**B**) Heatmap of growth factor expression profile from array assay. Expression levels are presented in red and green to indicate upregulation and downregulation. E-1, E-2 and C-1, C-2 refer to the duplicate measurement of the EF groups and Ctrl groups. The five most remarkably increased factors (compared with the ctrl group) are marked. (**C**) The optical density of the growth factor level is presented as fold change relative to NEG and compared with that of the control group. N = 3, n = 3. Data were compared using Mann–Whitney test: * *p* < 0.05 compared with the control (no EF).

To identify HaCaT-secreted human growth factors related to the regulation of wound healing, we performed an antibody-based human growth factor array assay after 7 days of EF exposure (Figure 5A). The ratios of growth factor levels in the EF groups compared with those in the control groups are presented as a heat map (Figure 5B). Significant increases in several human growth factors, such as GM-CSF (granulocyte-macrophage colony-stimulating factor) and PDGF (platelet-derived growth factor), which play important roles in wound healing and the tissue reconstructive process [29], could be observed. GM-CSF was the most abundant factor secreted by HaCaT cells after EF exposure (328.07% compared with non-exposed control cells), followed by FGF-7 (206.06%), and PDGF-AB (167.31%) (Figure 5C).

### 3.5. Erk1/2 Is Crucial for Responding to the Stimulation of EF

**Figure 6:**
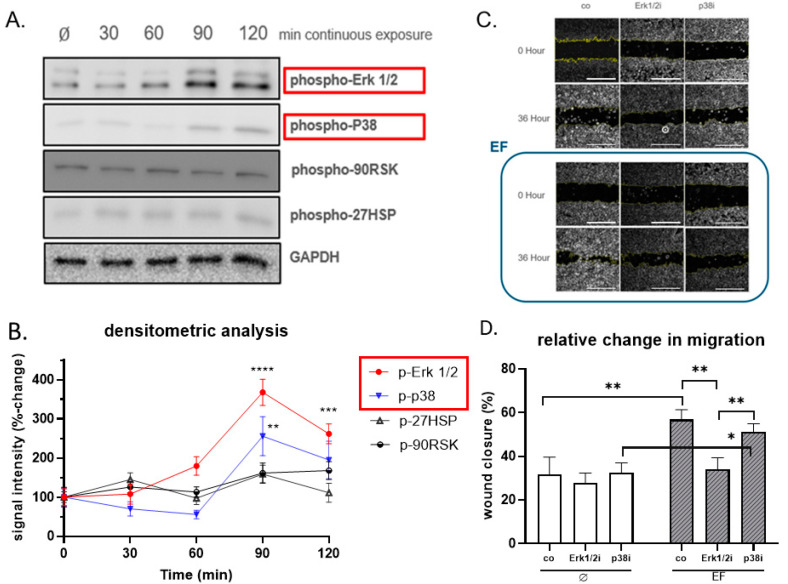
Regulation of Erk1/2 and P38 by EF. (**A**) Western blot signal for phospho-Erk1/2, phospho-p38, phospho-90RSK, and phospho-HSP27 in HaCaT cells 30, 60, 90, and 120 min after exposure to an EF of 200 mV/mm. The expression of GAPDH was used as a loading control. The densitometry readings/intensity ratio are provided in Supplementary Figure S1 and Table S1. Original Western blot images are shown in Supplementary Figure S2. (**B**) Protein levels were quantified by densitometric analysis of the Western blot via ImageJ Software. N = 3, n = 3. Data were compared using one-way ANOVA followed by Dunnett’s test: ** *p* < 0.01, *** *p* < 0.001, **** *p* < 0.0001. To explore the Erk1/2 and p38 activation in EF-induced migration, HaCaT cells were cultured with and without exposure to the EF in the presence and absence of inhibitors of Erk1/2 (U0126) and p38 (SB203580) signaling. (**C**) Image of scratch assay (scale bar = 400 μm) and (**D**) quantification of scratch assay. The hatched bars show EF-stimulated wound closure. N = 3, n = 3. Data were compared using non-parametric two-way ANOVA followed by Tukey’s multiple comparison test: * *p* < 0.05, ** *p* < 0.01.

To further examine the possible molecular target of EF for stimulation of HaCaT cells, different time points (30, 60, 90, and 120 min after exposure to the EF) were chosen to investigate the phosphorylation of signaling molecules via Western blot. The signals for phospho-Erk1/2 showed a significant rise (approx. +360%, *p* ≤ 0.0001) following EF exposure with a signal peak at 90 min; phospho-p38 showed a similar pattern of the signal changes. No pronounced differences in signals for phospho-90RSK and phospho-HSP27 could be observed following EF exposure (Figure 6A,B).

To determine the role of the EF-induced phosphorylation of Erk1/2 and p38 in migration, HaCaT cells were pretreated with specific MAP kinase chemical inhibitors for Erk1/2 (U0126) and p38 (SB203580). As presented in Figure 6C,D, the migration of HaCaT induced by the EF was significantly inhibited by the specific inhibitors of Erk1/2 signaling; however, the inhibition of p38 signaling did not show an influence on migration. Collectively, the results revealed that the EF enhanced the migration of HaCaT cells by phosphorylating Erk1/2.

## 4. Discussion

It has been known for more than 150 years that EF signals exist within the extracellular space of organisms [30]. The roles played by these electrical signals have been demonstrated in physiology, tissue regeneration, and pathology [31,32]. Dynamic extracellular electrical signals, such as action potentials, have been the earliest discovery and are the most studied by scientists [33]. Various research accomplishments (electrophysiological diagnosis, etc.) have been successfully translated into routine clinical procedures; however, the concurrent steady electrical signals associated, for example, with skin wounds have mostly been neglected [1]. Within the last three decades, an increasing number of research studies have confirmed that the steady electrical signals called the TEP may play a key role in cell biology, such as division, migration, and differentiation [32,34]. Furthermore, clinicians and physiologists have realized that the TEP may be critical for electrical signaling in the central nervous system, [35] tumorigenesis, [9] and wound healing [36].

Despite the growing clinical use of EFs for pain relief [37] and wound healing [6], we have little understanding of the precise underlying mechanisms of action. Specifically, there are limited in vitro experiments that have examined the relationship between EF application and wound healing in the skin. One of the main reasons is that major limitations exist in the conventional setup for this kind of in vitro study, such as the complexity and instability of existing models and the difficulty of translating EFs to multi-well plate experiments. In the present study, the experimental setup used to investigate the effect of EF on the migration, proliferation, and function of HaCaT cells represents a significant improvement over pioneering efforts [12,13,14,15]. A 6-well plate was used as the main body of an experimental setup, ensuring that identical EF signals could be received by up to four wells. The materials used to build the setups are easily attainable and affordable by any laboratory, increasing the feasibility of the experiments.

To verify cell proliferation enhanced by the EF, the EF-induced DNA synthesis, protein synthesis, and mitochondrial activity improvement of HaCaT cells were evaluated, and the results suggest a clear induction of cell proliferation in EF-exposed cells in a time-dependent manner. This observation agrees with a study by Hartig et al. [10] who investigated EFs, showing an enhancement in proliferation and alkaline phosphatase activity in osteoblasts compared with those of controls. Keratinocytes are one of the most important cell types in human skin and are primarily responsible for the epithelialization phase of wound healing [38]; moreover, keratinocyte proliferation is essential for wound healing [39].

According to our results, the EF showed a marked upward trend of HaCaT cell proliferation from day 1 to day 7, suggesting a potential therapeutic strategy to promote wound healing via the EF stimulation of cell proliferation in the clinic.

In this study, we demonstrated a significant improvement in the expression of healing-related growth factors induced by EF. Human growth factors play a critical role in the regulation and coordination of tissue reconstruction and wound healing processes, suggesting combined growth factor and electrical stimulation therapy as a potential clinical application. The present study demonstrated that the expression level of numerous growth factors, such as GM-CSF, PDGF, FGF-7 (fibroblast growth factor 7), etc., was markedly upregulated. GM-CSF, also known as colony-stimulating factor 2 (CSF2), is a glycoprotein-based growth factor that has essential functions during the process of wound healing [40,41]. It is synthesized by keratinocytes, fibroblasts, and many other cellular components that participate in wound healing [42,43]. GM-CSF enhances keratinocyte proliferation and migration, which favors the process of the re-epithelialization and reconstruction of the epidermal layers [44,45]. PDGF is produced by keratinocytes, vascular endothelium, fibroblasts, etc., and it comprises a family of PDGF-AA, AB, BB, CC, and DD, which have all been implicated in wound healing [46,47,48]. PDGF can effectively activate migration as well as proliferation in fibroblasts and the monocyte/macrophage systems, i.e., it is a critical activator for tissue repair. In fact, PDGF has been approved by the FDA (United States Food and Drug Administration) for the clinical treatment of chronic wounds [49]. FGF-7 is a member of the EGF family, which is secreted by keratinocytes, smooth muscle cells, mast cells, etc. [46,50,51], and a large number of studies have confirmed that FGF-7 plays a vital role in wound site re-epithelialization [52]. Moreover, FGF-7 promotes neovascularization via the activation of mitosis in vascular endothelial cells [53], also closely associated with wound healing. During our research on HaCaT cells exposed to EFs, up-regulation of growth factors involved in various stages of the tissue repair process was observed. Therefore, EF application in wound healing may hold promise for clinical application.

Overall, the increase in cell migration and proliferation, as well as the upregulation of growth factors, indicated that the wound healing ability of HaCaT cells increased after EF stimulation. However, it is still unclear how the EF signal activates intracellular signaling pathways. There is little evidence showing that EF- and EMF-induced cellular responses (e.g., migration, proliferation, and function) are regulated by multiple signaling pathways, and the MAPK (mitogen-activated protein kinase) signaling pathway is widely discussed [54,55]. In addition, as a common protective response, the upregulation of phospho-HSP27 (heat shock protein 27) can serve as an early biomarker of cellular stress [56]. Thus, in this study, we focused on the phosphorylation-level changes of phospho-Erk1/2, phospho-p38, and phospho-90RSK, three key molecules of the MAPK signaling pathway [57], and phosphorylation changes of HSP27 as well. We observed a significantly elevated level of phosphorylated Erk1/2 and p38 after EF exposure; meanwhile, the inhibition of Erk1/2 signaling with the chemical inhibitor U0126 prevented the enhancement of migration by the EF exposure, suggesting that the activation of the Erk1/2 signaling cascade is crucial for the facilitating effect of EF exposure on HaCaT cell migration. Moreover, in 2015, Yumoto et al. [55] confirmed that electromagnetic fields up-regulated PDGF expression via the Erk1/2 and p38 pathways, which matches the outcome of our human growth factor array assay. Furthermore, the EF exposure did not increase the phosphorylation of HSP27, which suggests that the EF may not provoke significant cellular stress, and this is in accordance with the results obtained by Shi et al. [56].

Naturally occurring in vivo EFs are an inherent attribute of wound sites. They have been thought of as initiators and integrators that regulate cells to function in wound healing. In the present study, the data demonstrate that EFs have a solid potential to improve wound healing by enhancing cell migration and proliferation and modulating a healing-related microenvironment, which provides an experimental basis for researchers and clinicians to apply EFs to accelerate wound healing. Although mechanistic studies still have a long path ahead, it will be exciting to see the development of “electrical therapy” in the field of regeneration and tissue engineering in the future.

## 5. Conclusions

Using a newly designed EF stimulation system on HaCaT cells, our study provided an experimental basis to demonstrate the cellular response to EFs, as evidenced by cell viability, cell proliferation, and human growth factor secretion related to wound healing. Furthermore, EFs may mediate HaCaT cell migration by activating the Erk1/2 signaling pathway. The clinical application of EF stimulation is still in an early stage; more fundamental research is still needed to explain the underlying mechanism. However, with in-depth basic and clinical research, EF stimulation may become an efficient tool for better tissue repair and wound healing.

## Data Availability

The datasets generated and/or analyzed during the current study are available from the corresponding author on reasonable request.

## Supplementary Materials

The following are available online at https://www.mdpi.com/article/10.3390/life11111195/s1, Figure S1: The densitometry readings/intensity, Figure S2: Original Western blot images are shown in Supplementary, Table S1: The densitometry readings/intensity ratio.

## Abbreviations

DMEM	Dulbecco’s modified eagle medium
DPBS	Dulbecco’s phosphate-buffered saline
EFs	Electrical fields
EF	Electrical field
EMFs	Electromagnetic fields
FDA	United States Food and Drug Administration
FGF-7	Fibroblast growth factor-7
GM-CSF	Granulocyte-macrophage colony-stimulating factor
HaCaT	Human keratinocyte cell line
MAPK	Mitogen-activated protein kinase
PDGF	Platelet-derived growth factor
SRB	Sulforhodamine B
TEP	Transepithelial potential
