# Supplementary material for: Direct Current Electrical Fields Improve Experimental Wound Healing by Activation of Cytokine Secretion and Erk1/2 Pathway Stimulation
Source: Life (Basel). 2021 Nov 5;11(11):1195. doi: 10.3390/life11111195 (PMC8625131; doi:10.3390/life11111195)
Supplement: Supplementary file 1 [file life-11-01195-s001.zip › life-1441598-supplementary/Supplementary Materials_Direct Current Electrical Fields Improve Experimental Wound Healing by Activation of Cytokine Secretion and Erk1_2 Pathway Stimulation.pdf]

life

MDPI

Supplementary Materials: Direct Current Electrical Fields Improve Experimental Wound Healing by Activation of Cytokine Secretion and Erk1/2 Pathway Stimulation

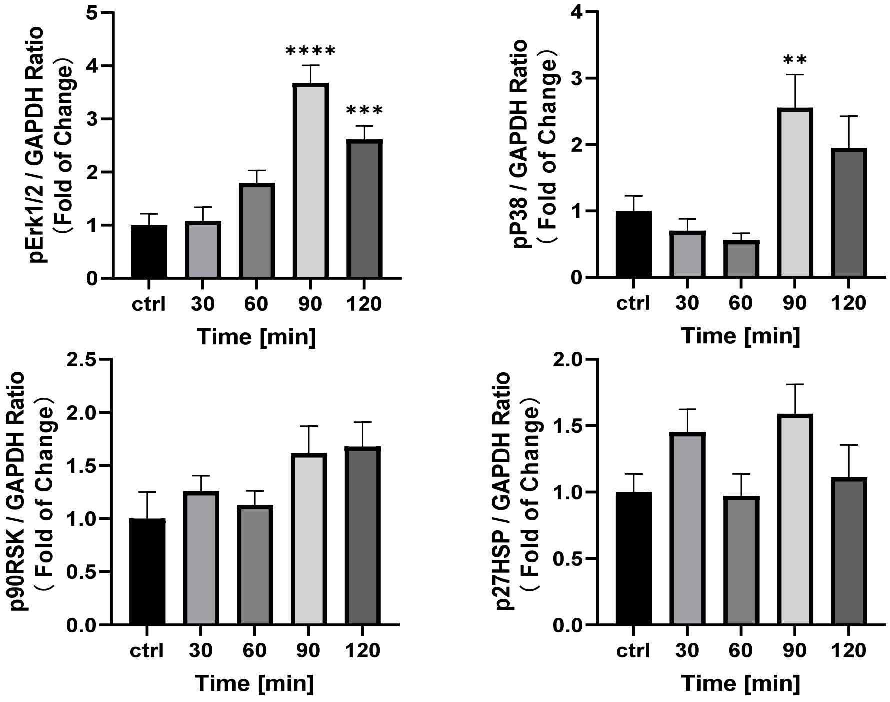

Figure S1. Quantitative analysis of phospho-Erk1/2, phospho-P38, phospho-90RSK, and phospho-27HSP abundance 30, 60, 90, and 120 min after exposure to an EF of 200 mV/mm. N = 3, n = 3. Data were compared by non-parametric two-way ANOVA followed by Tukey's multiple comparison test: ** $p < 0.01$, *** $p < 0.001$, **** $p < 0.0001$, as compared to the control (no EF).

Table S1. Densitometry Readings/intensity Ratio.

	pErk1/2 / GAPDH	pP38 / GAPDH	p90RSK / GAPDH	p27HSP / GAPDH
Ctrl	1.00000	1.00000	1.00000	1.00000
30min	1.07955	0.70071	1.25670	1.45103
60min	1.79286	0.55721	1.13016	0.97144
90min	3.67647	2.55619	1.61508	1.58866
120min	2.61600	1.94847	1.67817	1.11156

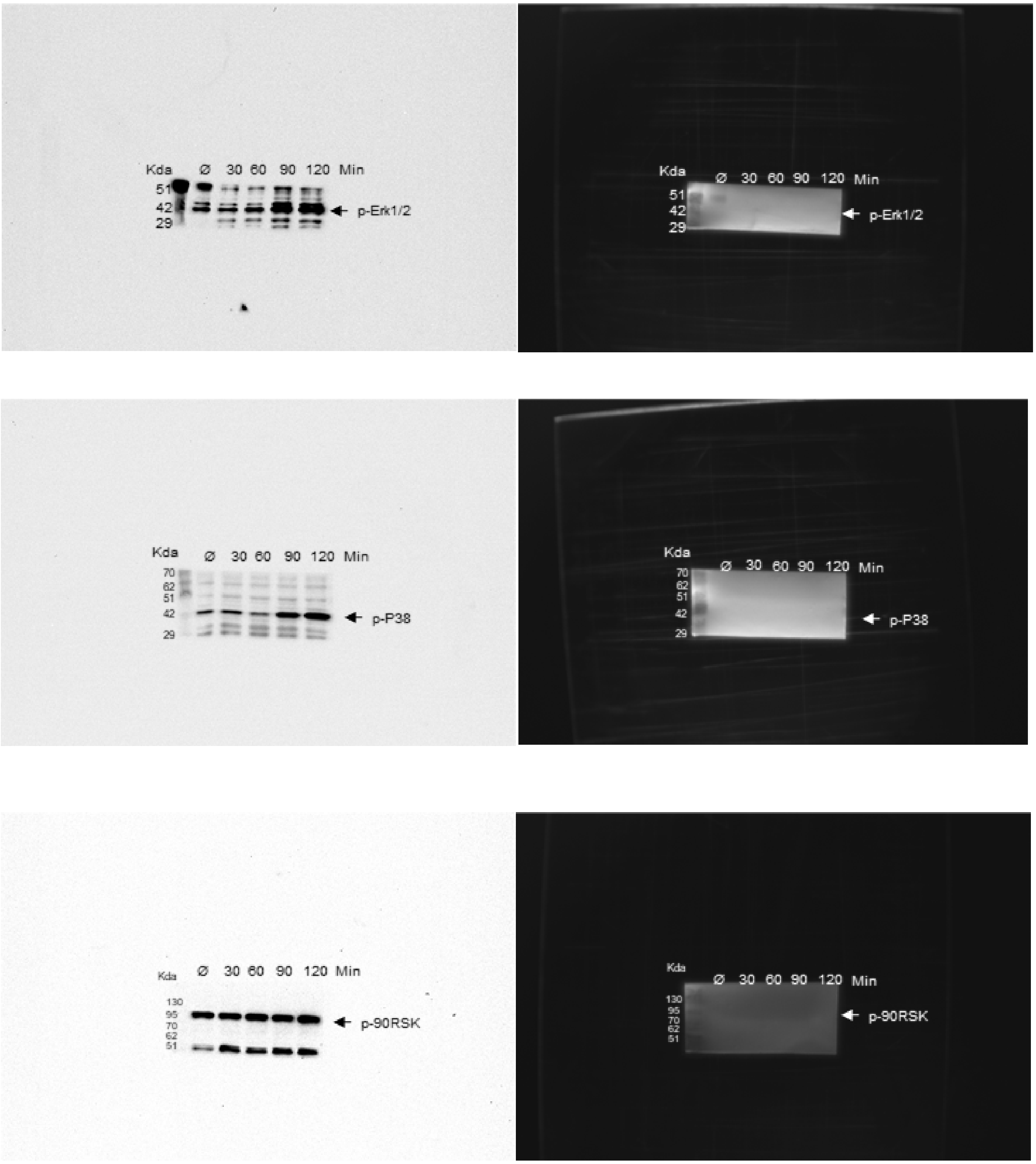
Kda
Ø 30 60 90 120 Min
51
42
29
p-Erk1/2
Kda
Ø 30 60 90 120 Min
51
42
29
p-Erk1/2
Kda
Ø 30 60 90 120 Min
70
62
51
42
29
p-P38
Kda
Ø 30 60 90 120 Min
70
62
51
42
29
p-P38
Kda
Ø 30 60 90 120 Min
130
95
70
62
51
p-90RSK
Kda
Ø 30 60 90 120 Min
130
95
70
62
51
p-90RSK

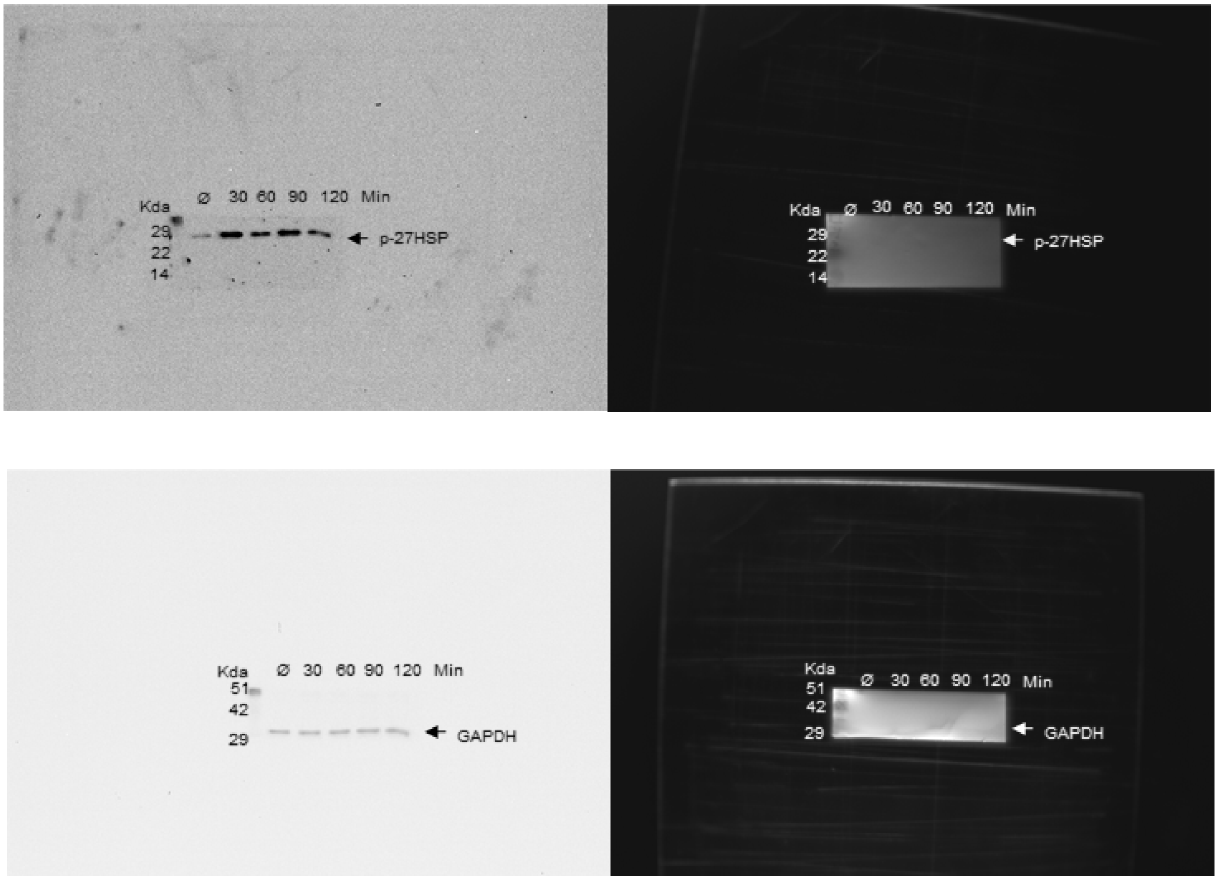

Figure S2. Regulation of Erk1/2 and P38 by EF Western blot signals (with white backgroud) and the corresponding plain images(with black background) for phospho-Erk1/2, phospho-p38, phospho-90RSK, and phospho-HSP27 in HaCaT cells 30, 60, 90, and 120 min after exposure to an EF of 200 mV/mm. The expression of GAPDH was used as a loading control.
