# Supplementary figures and images for: Direct Current Electrical Fields Improve Experimental Wound Healing by Activation of Cytokine Secretion and Erk1/2 Pathway Stimulation
Source: Life (Basel). 2021 Nov 5;11(11):1195. doi: 10.3390/life11111195 (PMC8625131; doi:10.3390/life11111195)

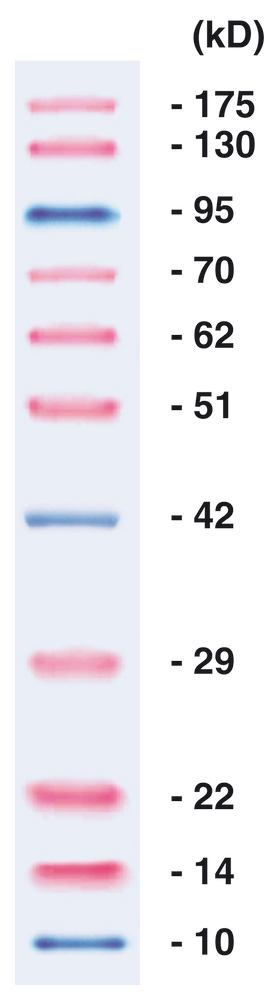

Supplement: Supplementary file 1 [file life-11-01195-s001.zip › life-1441598-supplementary/ROTI-Mark BI-Pink.jfif]
